# ALD-Induced Changes in Lithium Dynamics throughout Garnet-Type Solid-State Electrolytes: Insights from ^7^Li NMR *T*_1_ Relaxation

**DOI:** 10.1021/acs.jpclett.6c01110

**Published:** 2026-06-06

**Authors:** Michael K. Steinhoff, Davis Thomas Daniel, Shicheng Yu, Hermann Tempel, Rüdiger-A. Eichel, Josef Granwehr

**Affiliations:** † Institute of Energy Technologies - Fundamental Electrochemistry (IET-1), 28334 Forschungszentrum Jülich, 52428 Jülich, Germany; ‡ Material and Processes of Electrochemical Energy Storage and Conversion, RWTH Aachen University, 52074 Aachen, Germany; § Institute of Technical and Macromolecular Chemistry (ITMC), RWTH Aachen University, 52074 Aachen, Germany

## Abstract

Al_2_O_3_ atomic layer deposition (ALD) on garnet-type Li_6.4_La_3_Zr_1.4_Ta_0.6_O_12_ (LLZTO) powder has been shown to induce extensive lithium diffusion during layer formation, likely driven by proton-lithium exchange reactions. Here, we use ^7^Li MAS NMR and *T*_1_ relaxation data to study the influence on bulk lithium dynamics in LLZTO with respect to ALD coating thickness before high-temperature sintering. Considerable variation in relaxation characteristics was observed for the samples investigated, which could be traced back to diffusion phenomena occurring throughout the ALD process. These results suggest that surface modifications via ALD can significantly alter the lithium mobility of its host structure, underlining the necessity of material monitoring at different stages of cell production for a mechanistic understanding of various properties at device level. This work establishes spectrally resolved ^7^Li NMR *T*_1_ relaxation as sensitive tool for tracking ALD-induced changes in Li environments and ion dynamics relevant for systematic optimization of solid-state battery materials.

## Graphical abstract

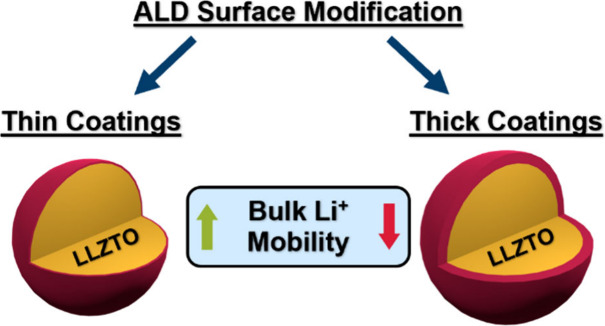

Thin-film technologies have proven to be a powerful tool for approaching crucial interface challenges associated with next-generation energy storage devices.^1–3^ Modification and functionalization of surfaces and interfaces between battery cell components via thin-films grown by vapor-phase deposition techniques are being readily applied to improve interface stability and resistance.^4–6^ In this context, the atomic layer deposition (ALD) process holds great promise by forming high-quality coatings and offering subnanometer control over film morphology and chemistry.^7,8^ Through sequential, self-limiting vapor-phase half-reactions, ALD can apply conformal coatings even on high-aspect-ratio surfaces, i.e., powder particles.^9–11^ For instance, ALD coatings on cathode powders, such as LiNi_*x*_Mn_*y*_Co_1–*x*–*y*_O_2_, improve capacity retention by reducing surface degradation and impeding transition metal dissolution into the electrolyte.^6,12,13^ Recently, this concept has also been applied to solid-state electrolyte (SSE) powders. Al_2_O_3_ coatings on Li_6_PS_5_Cl, Li_7_La_3_Zr_2_O_12_, and Li_1.3_Al_0.3_Ti_1.7_(PO_4_)_3_ were used to enhance ambient stability and ionic conductivity, or to improve the densification process and stability against lithium dendrite growth by engineering grain interfaces through precise control of ALD coating thickness.^14–17^

It was reported for cathode materials that Al_2_O_3_ coatings can undergo lithiation by extracting Li from the host structure during the ALD process, leading to the formation of a thin (∼2 nm) Li–Al–O-rich interface. This behavior was primarily attributed to temperature-driven diffusion, along with electrostatic interactions involving the trimethylaluminum precursor and reaction products arising from parasitic side reactions with residual surface species.^18–20^ We recently found that Al_2_O_3_ coated on the superionic conductor Li_6.4_La_3_Zr_1.4_Ta_0.6_O_12_ (LLZTO) can undergo long-range Li diffusion over several nanometers, possibly as a result of proton-lithium exchange reactions, acting as additional driving force. We proposed that diffusion of H^+^ embedded into the growing ALD layer in combination with the H_2_O-atmosphere during the ALD subcycle, causes extensive migration of Li^+^ into the ALD layer, resulting in a compositionally graded Li–Al–O coating.^17^ However, protonation is expected to significantly affect Li dynamics in LLZTO.^21^ Yet, it remains unclear whether this effect is confined to the surface, as expected for a core–shell structure with negligible shell influence on the core, or whether it also alters the bulk lithium dynamics of the garnet lattice. As this will be of great importance for understanding subsequent processing and electrochemical performance, precise analysis of Li environments and ion dynamics is required. Nuclear magnetic resonance (NMR) spectroscopy represents a versatile technique for probing structural characteristics and local environments of lithium inside solid-state battery materials.^22,23^ In addition, *T*_1_ relaxation experiments combined with inverse Laplace transform can offer insights into the dynamics of Li^+^ ions in multiphase systems.^24,25^ For instance, Schleker et al. demonstrated that spectrally resolved *T*_1_ measurements can sensitively detect changes in the bulk properties of Li_4_Ti_5_O_12_ arising from surface interactions with various liquid electrolytes.^26^

In this work, ^7^Li magic angle spinning (MAS) NMR and spectrally resolved *T*_1_ relaxation measurements were used to investigate changes in lithium dynamics throughout LLZTO powders induced by application of Li–Al–O ALD coatings of varying thickness. The material obtained directly after the ALD step was studied, without further processing for battery applications, to gain direct information about the influence of the coating step isolated from phenomena associated with the subsequent high-temperature sintering process, such as multiphase formation or grain growth. Laplace inversion was used to facilitate the separation of Li species differing in *T*_1_, and chemical shift variations were investigated by comparing spectra from different *T*_1_ regions. Subsequently, the results were correlated with the layer formation mechanism derived from high-resolution electron microscopy, ^27^Al MAS NMR, and X-ray photoelectron spectroscopy measurements previously reported for ALD-modified LLZTO.^17^

One-dimensional ^7^Li MAS NMR spectra were recorded from LLZTO powder samples with varying numbers of applied ALD deposition cycles (Figure 1). All spectra are dominated by a single resonance at a chemical shift of around 1.15 ppm which is in good agreement with the characteristic lithium sites of the garnet framework.^27,28^ Spinning side bands are visible at multiples of the MAS frequency and are marked accordingly. Furthermore, the first derivatives of the recorded spectra (see Figure 1c) show that there is no significant variation in chemical shift of the dominant resonance throughout the samples, as indicated by the red reference zero-point line, which corresponds to the peak maximum of the measured signals. However, a flattening in the slope of the curves, suggestive of changes in full width at half-maximum (fwhm) of the ^7^Li signal and possibly of an onset of weaker signals emerging with thicker Li–Al–O ALD coatings, can be observed in Figure 1c. The line broadening appears to be fairly symmetric. Furthermore, the absence of a distinct narrow central contribution is inconsistent with a core–shell configuration arising either from ALD-induced effects on ^7^Li in the outer region of the LLZTO particle, or from ^7^Li residing within the ALD coating. However, due to potential sensitivity limitations, line overlap, or exchange averaging, core–shell behavior cannot be explicitly excluded based on the present data. Nevertheless, as the major fraction of Li in LLZTO is located in regions distant from the surface, the observed line broadening suggests that the ALD coating significantly alters Li transport or local structure, or both, throughout the entire LLZTO particle.^29,30^

**Figure 1:**
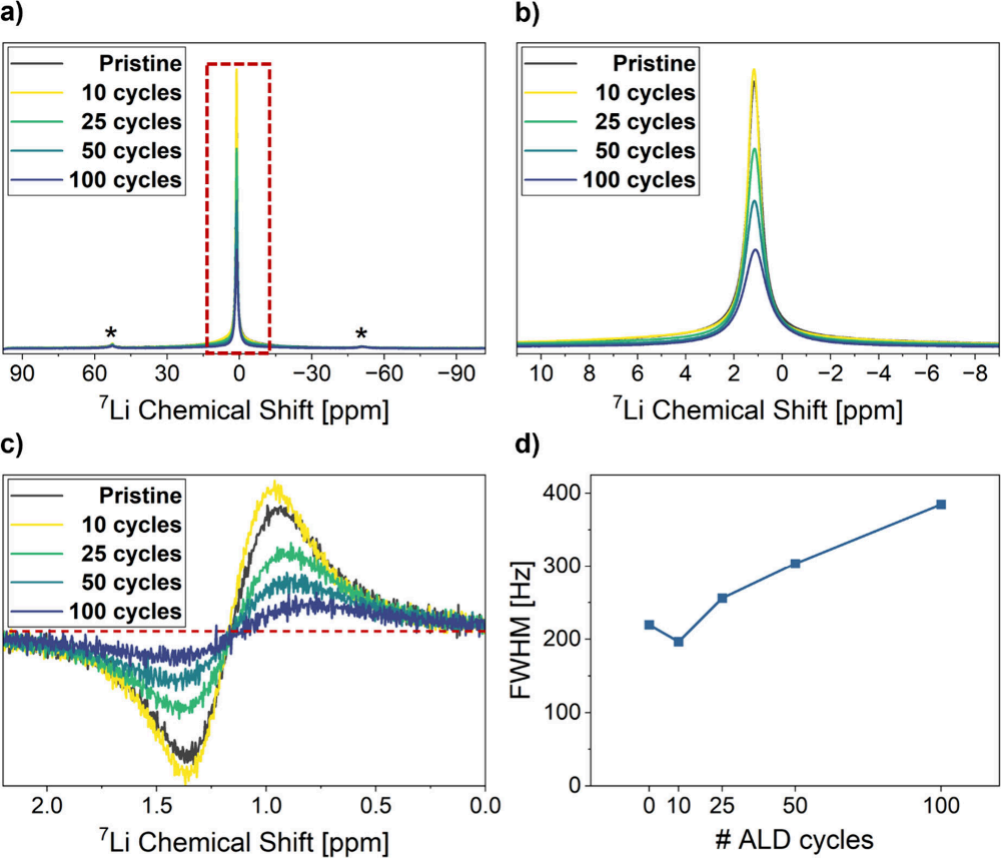
^7^Li MAS NMR spectra of pristine and ALD-coated LLZTO powder samples. (a) Overview spectra, with asterisks marking spinning sidebands. (b) Close-up of the same spectra. (c) First derivative of the recorded spectra in (a), with the red-dashed line marking the zero-point. (d) fwhm obtained from peak fitting of the resonances in (a).

The fitted fwhm’s with respect to the number of ALD cycles (Figure 1d) reveal, in general, a progressive signal broadening from a minimum of about 220 Hz for the pristine sample to a maximum of 385 Hz, apart from the 10-cycle sample, which shows a minor decline compared to pristine LLZTO. The observed fwhm evolution at a low number of ALD cycles could be indicative of ALD-induced variations in the population of lithium atoms occupying environments at the surface different from the well-ordered LLZTO host lattice, likely arising from reported phenomena such as the removal of residual surface carbonates or lithium incorporation into the growing ALD layer.^17,20^ While such surface alterations have a considerable impact on interface properties, their direct impact on bulk LLZTO, as observed by minor fwhm-variation, is limited yet non-negligible. In contrast, as the number of ALD cycles increases, the substantial fwhm-increase of the ^7^Li signal could imply that the mobility of lithium ions is notably affected in those samples. However, instead of homogeneous line broadening, an increasing distribution of isotropic chemical shifts may also cause larger fwhm values. As the spectra are masked by the garnet resonance, the origin of these changes remains inconclusive. For disambiguation and to obtain further insights related to the influence of the ALD procedure on the lithium-ion dynamics in bulk LLZTO, complementary *T*_1_ relaxation analysis was employed to separate motional from purely structural broadening effects (Figure 2).

**Figure 2:**
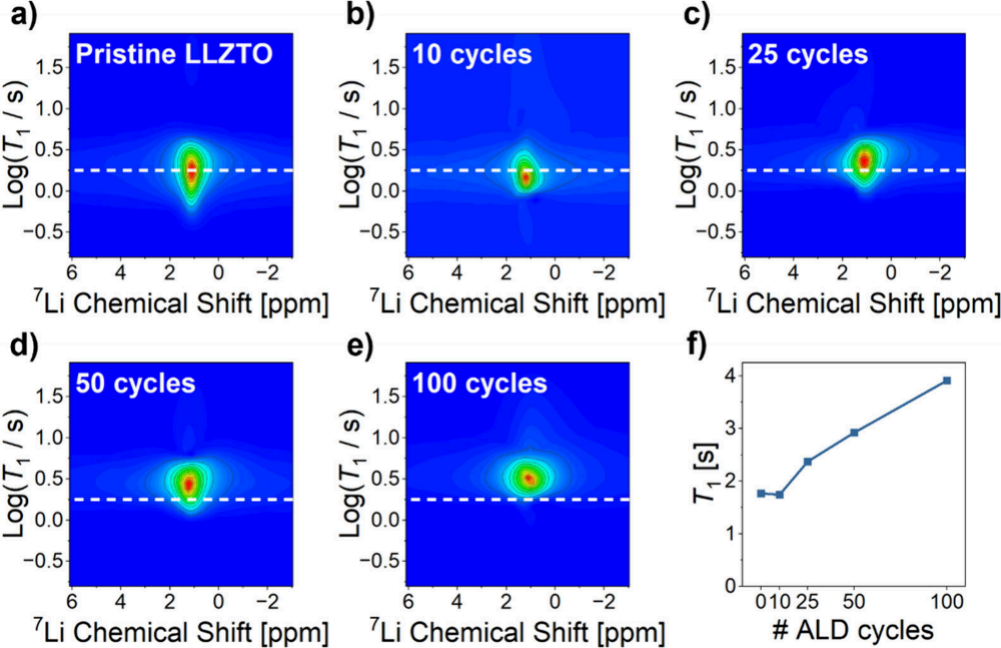
(a–e) Spectrally resolved ^7^Li *T*_1_ relaxation distributions in pristine and ALD-coated LLZTO samples. The white-dashed line marks the weighted average relaxation time of pristine LLZTO. (f) Weighted average *T*_1_ data obtained at the maximum of the spectral dimension with respect to the number of applied ALD cycles.

The spectrally resolved *T*_1_ distributions of ^7^Li in the LLZTO samples are illustrated in Figure 2a–e. As highlighted by the white reference line for the weighted average relaxation time in pristine LLZTO, the ALD process introduces changes in the *T*_1_ distribution, corresponding to variations in lithium-ion mobility of the material. The *T*_1_ distributions integrated along the spectral dimension of all the samples can be represented well by two contributions, a main mode (blue-shaded area) that shows the spectral features of the dominant resonance as identified by ^7^Li MAS NMR in Figure 1, and a slow mode (yellow-shaded area) at longer *T*_1_ (log­(*T*_1_/s) > 0.94) (Figure 3a). The main mode can be fitted well using two half-normal distributions, one for the part of the distribution with *T*_1_ shorter and one for *T*_1_ longer than the mode maximum. The width difference between these modes can be used as a qualitative measure for the skewness of the mode, and the mean of their width for the homogeneity of mobile Li (Figure 3b). The slow mode does not show a well-defined distribution. However, its integral, which could be indicative of contributions from lithium ions in secondary phases, hopping processes across intraparticle grain boundaries, immobilized Li, and the Li-containing ALD coating, can be estimated by subtracting the main mode fit from the overall *T*_1_ distribution. Figure 3c shows the fraction of the total signal of each data set taken up by the slow mode.

**Figure 3:**
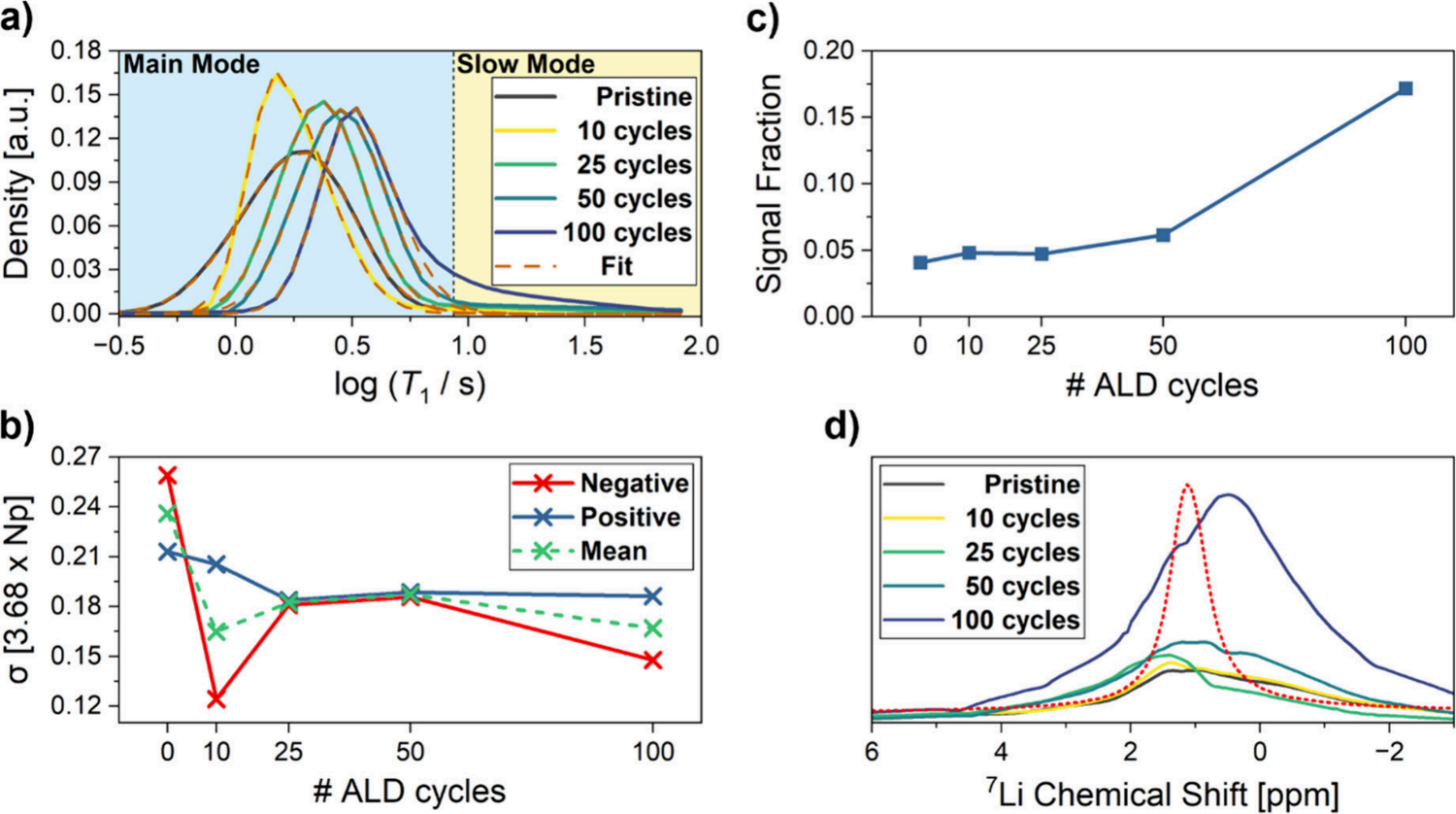
(a) ^7^Li *T*_1_ relaxation distributions for different ALD coating thicknesses, obtained by integrating along the spectral dimension, with fits (dashed lines) of the main modes using a negative and a positive half-normal distribution. Distributions are normalized to the same integral. (b) Width of the Gaussian fits of the main mode including the negative, positive, and mean half-normal distribution in units of logarithmic decades denoted by the Neper unit (Np). (c) Relative integral of the slow mode at long *T*_1_ vs ALD coating thickness. (d) ^7^Li NMR spectra of the slow *T*_1_ contribution (corrected by the main mode) for pristine LLZTO and ALD-coated samples normalized to the same integral of each full data set. The dashed line represents the main mode spectrum (pristine LLZTO), scaled down for visualization.

Compared to pristine LLZTO, overall relaxation becomes slightly faster for the 10-cycle sample. As more ALD layers are added, *T*_1_ increases by as much as a factor of ∼2 for 100 ALD cycles (Figure 2f). This behavior is in good agreement with the obtained trends in the spectral fwhm of the ^7^Li MAS NMR spectra in Figure 1d, confirming the link between structural modification of the LLZTO surface and lithium-ion mobility. The *T*_1_ relaxation time distributions of ^7^Li, integrated along the spectral dimension (Figure 3a), reveal variations in symmetry and fwhm for the main *T*_1_ mode throughout the investigated samples. In pristine LLZTO, a broad and slightly negatively skewed distribution, indicative of further relaxation modes or a distribution of environments with fast *T*_1_, can be seen. Through application of 10 ALD cycles, the shape of the *T*_1_ mode becomes considerably narrower and more asymmetric yet positively skewed compared to the pristine sample. However, both the positive and the negative half-normal distributions show a smaller line width than the pristine sample. Asymmetry is observed to reduce again with further increasing the number of applied deposition cycles, resulting in an almost log-symmetrical relaxation mode in 25- and 50-cycle samples. For 100 ALD cycles, the main relaxation mode becomes positively skewed again and increased slow-mode density can be seen at long *T*_1_ (Figure 3c). The corresponding slow-mode spectrum, which is broad and rather featureless in the pristine and 10-cycle samples, shows an upfield shift compared to the main resonance by ∼−0.4 ppm with a width of about 800 Hz (Figure 3d). Its intensity could already be seen building up in 50-cycle samples, which is attributable to the increasing volume fractions of ∼5.7 (50 cycles) and ∼10.9 vol % (100 cycles) that the ALD coating accounts for. This could further suggest that with increasing thickness, Li inside the coating resides in increasingly ordered environments with a defined chemical shift.

The *T*_1_ relaxation results could be interpreted in accordance with the proposed layer formation model in our previous publication^17^ to describe ALD-induced changes in bulk Li dynamics of the garnet structure (Figure 4a). In its pristine state prior to deposition, the width of the *T*_1_ mode represents a dynamically heterogeneous Li population in the sample with a broad lithium-ion mobility distribution (Figure 2a, Figure 3a). This can be attributed to several contributions. Bulk LLZTO contributes the main density of the relaxation mode, with variations in site occupancies of Li^+^, surface-near defects and surface residuals (i.e., Li_2_CO_3_, LiOH), characteristic of polycrystalline LLZTO. Thereby, paramagnetic defects and local high-mobility environments contribute to the fast wing of the relaxation mode, while Li incorporated in passivated surface regions or in possible side phases, as well as in the vicinity of grain boundaries shows slow *T*_1_. During the initial stages of the ALD process, the material changes drastically as two important phenomena are expected to occur. First, residual surface contaminants are removed through either precursor etching or parasitic side reactions.^19,20^ Second, based on the *T*_1_ data, one could assume that space-charge effects at the coating/LLZTO interface also play a significant role particularly for low ALD cycle numbers. Thermal vacuum annealing through the applied deposition temperature of 225 °C introduces surface defects, such as oxygen vacancies, while at the same time Li chemical potential gradients at the interface induce redistribution of Li through Li^+^/Al^3+^ interdiffusion. Limited to the subsurface, this results in the formation of a Li_6.4–3*x*_La_3_Zr_1.4_Ta_0.6_Al_*x*_O_12_ (Al-LLZTO) solid solution, annihilating surface defects and introducing Li-vacancies that act as diffusion channels with reduced activation barrier between the ALD coating and LLZTO. The synergy of these interface-related effects is expected to result in a relative homogenization of the structure, which is reflected in the narrowing of the *T*_1_ distribution in Figure 3a, leading to a mean enhancement of *T*_1_ relaxation. Since the deposition temperature is moderate compared to LLZTO sintering temperatures, thermal annealing is not sufficient to introduce notable variations in grain microstructure or bulk crystal structure, including intraparticle grain boundaries. Thus, the material changes are limited to the surface and subsurface, yet the observed change of the ^7^Li relaxation distribution suggests that surface modifications of the pristine LLZTO might also affect lithium-ion mobility distant from the surface, in analogy to observations for lithium titanate in contact with liquid electrolyte where no chemical reactions but only surface charge redistribution occurred.^31^ Here, the narrowing of the relaxation mode corresponds to a more homogeneous distribution of lithium-ion mobility over extended length and time scales, since *T*_1_ represents a weighted average of the relaxivity in the different environments sampled by a lithium ion within the time scale set by *T*_1_. Although both slow- and fast-relaxing Li contributions are reduced, the distribution remains positively skewed as intraparticle grain boundaries are not directly altered by the ALD process. Moreover, H^+^/Li^+^ exchange-driven diffusion of lithium into the growing ALD layer, facilitated by thermally enhanced H^+^ mobility, would introduce additional ^7^Li environments originating from the emerging Li–Al–O coating and protonated LLZTO.^17^ Since H^+^ is largely immobile at room temperature in LLZTO and Li^+^ has a low mobility in Li–Al–O, this effect is expected to reduce mobility and could contribute to the asymmetric distribution. However, a significant fraction of low mobility Li in an environment different from LLZTO should be visible in the ^7^Li MAS NMR spectrum. It follows that for thin coatings (10 cycles), the impact of the protonation remains limited to the surface and cannot adversely influence the bulk Li^+^ mobility of LLZTO. Consequently, relaxation in these samples is dominated by ALD-enhanced interface ion dynamics similar to other studied nanostructured conductor-isolator composite solid electrolytes.^32–35^

**Figure 4:**
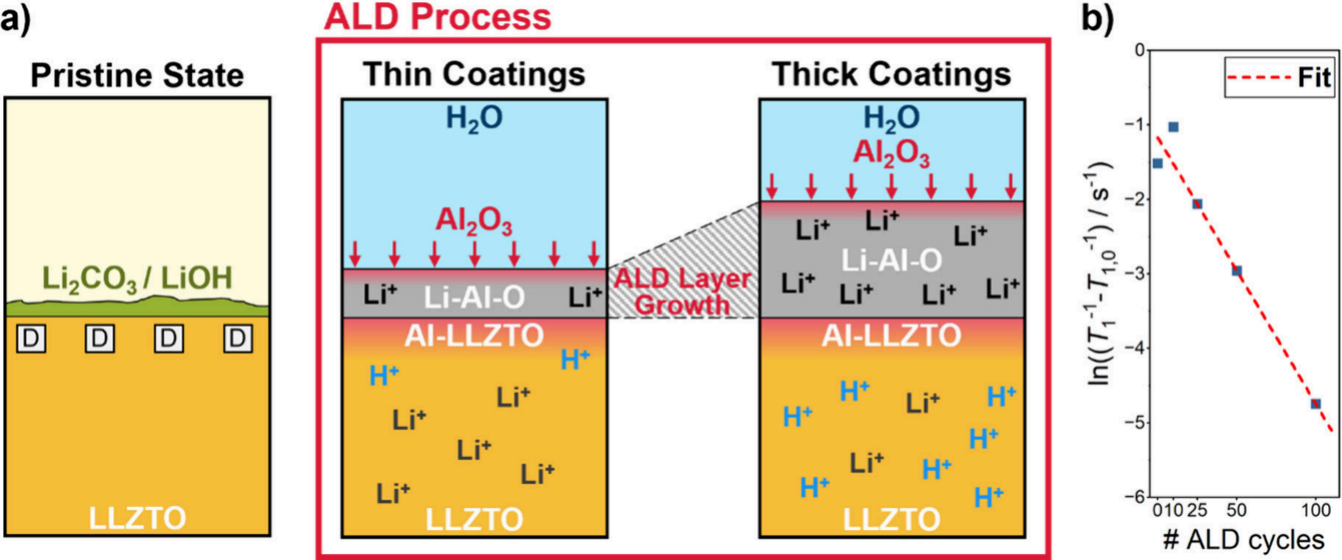
(a) Schematic illustration of the proposed changes occurring at the surface and the bulk of LLZTO during the Li–Al–O ALD process from correlation with the ^7^Li *T*_1_ data. (b) Linearized plot of ^7^Li *T*_1_ relaxation vs ALD cycle number. The three data points with the largest ALD layer were used to calculate θ_1/2_ ≈ 28.0 and *T*_1,0_ ≈ 3.30 s. The red line represents the calculated straight line.

For intermediate coating thicknesses (25/50 ALD cycles), the material structure appears to become more heterogeneous, with a more defined Li–Al–O coating evolving. Nevertheless, the ^7^Li *T*_1_ distribution becomes more symmetric, and for 25 ALD cycles, the slow mode in the distribution does not get more densely populated. This could imply an increasing ^7^Li population inside the Li–Al–O layer, yet near the interface to LLZTO. Percolation in LLZTO is maintained and the amount of completely immobile Li that is decoupled from mobile Li species is still low. This is also consistent with the slow mode spectra in Figure 3d, in which the 25-cycle sample shows a significant change. Compared to pristine LLZTO and 10 ALD cycles, a signal around 1.4 ppm increases, while lower intensities are observed for the upfield region of the slow mode. However, at the same time, the average lithium-ion mobility decreases, which is expressed in a peak shift in the *T*_1_ distributions in Figure 3a, as well as a resonance broadening in the ^7^Li MAS spectra (Figure 1) from 10- to 25/50-cycles. Such a trend would be consistent with an onset of H^+^/Li^+^ exchange within LLZTO during ALD. Li^+^ ions in sites with low activation barriers get exchanged first. At the same time, very slowly exchanging or immobile H^+^ occupy octahedral sites inside the garnet lattice, leading to lattice distortions and disrupting lithium migration pathways.^36–38^ In combination with generally decreasing concentrations of Li^+^ in LLZTO, the negative influence of ALD surface modification is reflected for the first time in the overall reduction of the lithium-ion mobility of the bulk structure.

Finally, with thick coatings (100 ALD cycles), pronounced H^+^/Li^+^ exchange in bulk LLZTO is most likely the reason for further increasing *T*_1_ relaxation times. The narrow, still fairly symmetric main *T*_1_ relaxation mode in Figure 3a indicates that protonation, facilitated by the extended thermal annealing, is not limited to the surface region of the LLZTO and affects lithium-ion mobility well within the LLZTO bulk, which would be consistent with reported mixed-ionic conduction in LLZTO at elevated temperatures.^39^ At the same time, the slow *T*_1_ relaxation mode starts to noticeably increase. The difference of its intensity compared with the value of the samples with lower numbers of ALD cycles implies that more than 12% of the overall Li has been exchanged into the increasingly well-defined Li–Al–O coating. This is corroborated by noticing that the species contributing to the slow *T*_1_ mode show a distinguishable chemical shift compared to ^7^Li in LLZTO (Figure 3d). Nevertheless, an in-depth mechanistic interpretation of ^7^Li *T*_1_ relaxation may represent not only the dynamics of the Li^+^ ions, but also that of nearby H^+^ ions. To support such an analysis, multinuclear (^7^Li, ^1^H) NMR experiments in combination with atomistic simulations are required not only for the autocorrelation function of the observed nucleus, but also for the autocorrelation function of the relaxation-inducing interactions.^40^

As lithium-ion mobility progressively decreases with continuing ALD-induced protonation, the proton-lithium exchange reaction is expected to continuously slow down, which results in a lithium gradient throughout the Li–Al–O coating, eventually reaching an equilibrium state, beyond which further ALD growth is expected to form pure Al_2_O_3_ with protons accumulating at the coating/LLZTO interface. However, up to 100 ALD cycles the mean *T*_1_ is still increasing, demonstrating that such an equilibrium state has not yet been fully established, which is attributable to the thermally enhanced diffusion kinetics. In this regard, quantitative evidence for ALD-modified Li^+^ ion dynamics in bulk LLZTO could potentially be provided through lithium diffusion measurements via pulsed field gradient (PFG) NMR. However, previous experiments on LLZTO powders revealed diffraction patterns attributable to hindered diffusion at physical barriers, indicating that the assumption of homogeneous diffusion used in standard PFG NMR analysis is not fulfilled by the powder morphology of the sample. Sufficient densification might reduce those effects, but as the garnet-type material cannot be adequately densified without high-temperature sintering, the diffusion coefficients extracted from PFG NMR measured on the samples investigated in this work may be systematically distorted and difficult to interpret quantitatively. Therefore, reliable PFG NMR measurements would require dedicated methodological developments and advanced modeling. Instead, an *ad hoc* model describing *T*_1_ relaxation as a function of the ALD coating thickness θ can be attempted as semiquantitative trend analysis. By assuming that relaxation of the pristine LLZTO is dominated by a single motional correlation rate ρ_0_ = τ_c_^–1^, in which τ_c_, for fast ion conductors, may be considered as the ion jump time constant,^41^ *T*_1_ can then be approximated as

1 $$T_{1}^{-1}=c\rho_{0}+T_{1,0}^{-1}$$

where *c* is a constant and *T*_1,0_ is a process that eventually takes over if ρ_0_ approaches zero, e.g., as observed by going to cryogenic temperatures.^42^ Assuming a first-order variation of this rate with ALD coating thickness, the solution to this equation,

2 $$\rho \left(\theta \right)=\rho_{0}\;\exp\left(-\frac{\theta }{\theta_{1/2}}\right)$$

with half-thickness θ_1/2_, implies an Arrhenius-type behavior for *T*_1_^–1^-*T*_1,0_^–1^. By fitting *T*_1,0_ and plotting ln (*T*_1_^–1^-*T*_1,0_^–1^))­vs.θ the negative slope represents $\frac{1}{\theta_{1/2}}$ which may serve as a comparative descriptor for assessing how strongly a given ALD protocol perturbs Li dynamics in LLZTO. Figure 4b shows a calculation of slope, intercept and *T*_1,0_ using the three data points with highest number of ALD cycles. Since the initial ALD cycles led to a surface modification of LLZTO before a stable layer evolved, the present data set only contains three data points within the range of θ where such a model may be faithful, hence the three parameters of the model are fully determined with three points and residuals cannot be used to assess its validity. Nevertheless, extrapolation to lower θ is not beyond reasonable expectations. Moreover, the obtained *T*_1,0_ ≈ 3.30 s is in line with values found for superionic conductors,^42^ albeit not implying that complete inhibition of lithium-ion mobility would be expected. The obtained half-thickness of 28 ALD cycles not only indicates that, for the present material and ALD protocol, a careful adjustment of the cycle number is crucial to avoid overly sacrificing Li mobility, but it also provides a phenomenological framework to gauge against other performance-relevant aspects of the ALD procedure.

Taken together, ^7^Li NMR *T*_1_ relaxation investigations were used to examine the effect of ALD-based surface functionalization on the bulk Li dynamics of LLZTO. The results revealed pronounced variations in bulk lithium-ion mobility, which are in good agreement with the phenomena related to the previously proposed layer formation mechanism via proton-lithium exchange reactions. The *T*_1_ relaxation data suggest that ALD-modified LLZTO cannot be adequately described by a simple, noninteracting core–shell model. Instead, the coating process perturbs Li dynamics over a substantially larger fraction of the particle volume even before the subsequent sintering step. As the LLZTO surface is being modified by removing surface residuals and defects, by local interdiffusion, as well as by proton-lithium exchange-induced lithium migration into the growing Al_2_O_3_ layer, changes in the relaxation of bulk Li in LLZTO of the investigated samples were detected. While the former two effects, together with limited protonation at low coating thickness, appear to transiently homogenize the Li dynamic landscape and modestly accelerate average *T*_1_ relaxation relative to pristine LLZTO, considerable H^+^/Li^+^ exchange seems to become the dominating factor with increasing coating thickness, ultimately resulting in slower *T*_1_ relaxation. Furthermore, these findings underline that ALD-generated surface modification disturbs the overall Li balance of the LLZTO. This holds important implications for the future design of ALD coatings on SSEs as molecular-level analysis techniques are required to also investigate the effect on the bulk lithium behavior and local coordination environments of the host structure. We therefore propose ^7^Li NMR *T*_1_ relaxation experiments in combination with inverse Laplace transform analysis as a suitable strategy to closely monitor protonation of ALD-coated SSEs for optimization of high-performance all-solid-state battery materials.

## Experimental Methods

Powder atomic layer deposition was performed on commercial Ampcera Li_6.4_La_3_Zr_1.4_Ta_0.6_O_12_ (average particle size: ∼1.4 μm) from MSE Supplies LLC, using a Picosun R-200 Advanced ALD-system from Applied Materials, Inc., at Nanexa AB, Sweden, in stop-flow mode. After a stabilization period of 90 min at 225 °C (denoted as pristine LLZTO), trimethylaluminum (EpiValence) and deionized water were used as precursor material with nitrogen as inert gas carrier. Each ALD subcycle consisted of 20 precursor pulses (1 s) with subsequent 30 s soaking time and inert gas purging. The number of applied ALD cycles was varied (10, 25, 50, 100 cycles), which resulted, with a growth per cycle of ∼2.4 Å, in coating thicknesses of 3.8 ± 0.2 nm, 6.8 ± 0.1 nm, 12.9 ± 0.4 nm, and 24.5 ± 0.8 nm, respectively.^17^ Consequently, the volume fraction of the ALD coating amounts to approximately 1.7, 2.9, 5.7, and 10.9 vol %.

For solid-state MAS NMR experiments a Bruker AVANCE NEO spectrometer (18.78 T), which was equipped with a 3.2 mm triple-resonance H/X/Y CPMAS probe, was used. With operating frequencies and a MAS frequency of 310 MHz and 16 kHz, respectively, as well as a 90° excitation pulse length of 2.2 μs, ^7^Li spectra were obtained. The sample temperature was kept at 25 °C. TopSpin 4.1.0 was used for data processing, e.g., phase and baseline corrections. The spectra were externally referenced to LiF at −1 ppm.^43^ Fitting was conducted using Lorentzian functions in Origin to extract peak position and fwhm. *T*_1_ relaxation measurements were performed using an inversion recovery sequence with 11 recovery delays and 8 scans were acquired point with a relaxation delay of 10 s. The relaxation data was analyzed by an inverse Laplace transform using the ILTpy library, which was run on Python 3.12.7.^44^ For inversion, the 2D relaxation vs spectral data and an exponential kernel was used. Only the relaxation dimension was inverted, but both dimensions were regularized. An identity kernel was used in the noninverted dimension, and a non-negativity constraint was not used for the inversion. The *T*_1_ distributions were calculated using logarithmically spaced output sampling ranging from 10^–1.5^ to 10^4^ s with 80 points. The parametrization for the inversion is described elsewhere.^45^ Postprocessing of *T*_1_ distribution data was carried out using a Gaussian fit function including a negative and positive half-normal distribution with GNU Octave 11.1.0.

## Data Availability

The data is available upon reasonable request from m.steinhoff@fz-juelich.de.
